# Psychiatric face of COVID-19

**DOI:** 10.1038/s41398-020-00949-5

**Published:** 2020-07-30

**Authors:** Luca Steardo, Luca Steardo, Alexei Verkhratsky

**Affiliations:** 1 University Magna Graecia, Catanzaro, Italy; 2 Sapienza University Rome, Rome, Italy; 3 Fortunato University, Benevento, Italy; 4 Faculty of Biology, Medicine and Health, The University of Manchester, Manchester, M13 9PT UK; 5 Achucarro Center for Neuroscience, IKERBASQUE, 48011 Bilbao, Spain; 6 Sechenov First Moscow State Medical University, Moscow, Russia

**Keywords:** Psychiatric disorders, Molecular neuroscience, Psychiatric disorders, Molecular neuroscience

## Abstract

The Coronavirus Disease 2019 (COVID-19) represents a severe multiorgan pathology which, besides cardio-respiratory manifestations, affects the function of the central nervous system (CNS). The severe acute respiratory syndrome coronavirus 2 (SARS-CoV-2), similarly to other coronaviruses demonstrate neurotropism; the viral infection of the brain stem may complicate the course of the disease through damaging central cardio-respiratory control. The systemic inflammation as well as neuroinflammatory changes are associated with massive increase of the brain pro-inflammatory molecules, neuroglial reactivity, altered neurochemical landscape and pathological remodelling of neuronal networks. These organic changes, emerging in concert with environmental stress caused by experiences of intensive therapy wards, pandemic fears and social restrictions, promote neuropsychiatric pathologies including major depressive disorder, bipolar disorder (BD), various psychoses, obsessive-compulsive disorder and post-traumatic stress disorder. The neuropsychiatric sequelae of COVID-19 represent serious clinical challenge that has to be considered for future complex therapies.

## Introduction: infectious pandemics as a risk factor for psychiatric diseases

Human civilisation has always co-existed with parasitic forms of life represented by bacteria and viruses that invariably took the toll of life. When social, biological and economic factors aligned, the infections became widespread reaching the level of pandemic, which caused massive death and misery; pandemics shaken the foundations of society and turned the course of history and mindset of humanity. The typhoid fever devastated Athens in 490 BC thus giving the military society of Sparta upper hand in Peloponnesian war, the Plague of Justinian doomed the reincarnation of Roman empire, while the Black Death, caused by *Yersinia pestis* that killed a third of population of Europe, instigated tectonic changes in economic relations that ultimately disposed of serfdom and feudalism and laid foundations of Renaissance. The last global epidemic of Spanish flu responsible for 20–50 millions deaths has coincided with First World War, internecine conflicts and birth of bolshevism, which all together brought the greatest confusion to mankind. Movements of great masses of soldiers from the US brought the H1N1 influenza A virus to Europe; disruption of the health services, poor hygiene associated with movements of people, devastations of war and malnutrition all sparkled the superinfection with unusually high death toll^1^.

All major pandemic, being associated with severe environmental stress, affected human way of thinking and human psychological health. Systematic studies aimed at identifying pathogenetic mechanisms responsible for the onset of psychiatric diseases following viral epidemics begun in 19 century. The eminent English doctor, Henry Holland in 1839 proclaimed that the flu was responsible “of featured impairments of mental functions almost in the same ratio of the body ……. and that the behavioural alterations were not comparable to those secondary to other fevers”^1^. Eighty years later Karl Menniger confirmed the association between viral infection and psychiatric morbidity: “one hundred cases of mental disease associated with influenza in the recent pandemic have been studied at the Boston Psychopathic Hospital. The variety of mental disturbance manifested is wide… they are readily classifiable into four groups: delirium, dementia praecox, other psychoses, and unclassified. Of these, dementia praecox is the largest group numerically” 2.

Over the years the accumulated clinical evidence has strengthened our knowledge of psychiatric features of cerebral disease. In the past few decades the interest in the putative aetiologic role of viruses has gradually enhanced to enclose not only the organic mental disorders induced by acute viral encephalitis and the slow viral infections of the central nervous system (CNS) but also to encompass the so-called functional psychiatric diseases such psychosis, depression and bipolar disorder (BD). It has became universally acknowledged that combination of systemic infection, viral neurotropism and environmental stress facilitates or even induces development of psychiatric pathologies that exacerbate the course of pandemic and present a significant therapeutic challenge.

## Neurotropism of coronaviruses

The Coronavirus Disease 2019 (COVID-19) pandemic revives a long-forgotten challenge for humanity that lived in (illusionary) mass infection free environment. Grappling with the uncertainties of a newly emerged disease, against which neither vaccine nor effective treatment protocol exists, the mankind will likely subsist in a new reality for months if not years before implementation of a global remedy. How the virus interacts with our body and which are the pathophysiological scenarios for acute phase of the disease and long-lasting outcomes are the critical questions to be addressed to identify medical strategies.

The COVID-19 results from the infection with a novel coronavirus that was first identified in China following an initial outbreak in 2019^2^. This coronavirus, named as severe acute respiratory syndrome coronavirus 2 (SARS-CoV-2) belongs to group 2B of β-coronavirus family^3^. The SARS-CoV-2 is recognised as the seventh component of the coronavirus family and has been included in the orthocoronavirinae subfamily^4^. Coronaviruses are single-stranded RNA viruses generally related to respiratory illness; they also (albeit less frequently) may instigate gastrointestinal and neurological disorders in a wide variety of mammals and birds. The coronaviruses have high rates of mutation and recombination as well as a propensity of cross-species transmission^5^. The SARS-CoV-2 enters the cell following binding to plasmalemmal ACE2 enzyme with subsequent endocytic internalisation^6,7^. The primary targets for the virus are represented by epithelial cells of the lungs and gastrointestinal tract. Endocytosis of the ACE2-virus complex also leads to a depletion of plasmalemmal pool of ACE2 with consequent reduction in conversion of Angiotensin II to Angiotensin 1–7; the latter peptide possesses marked anti-inflammatory properties^8,9^ and the reduction of Arg 1–7 significantly contributes to lung failure and the massive occurrence of pulmonary fibrosis described in patients with COVID-19^10^. Whether SARS-CoV-2 could penetrate cells through alternative routes remains unclear, although in contrast to other coronaviruses, SARS-CoV-2 does not bind to plasmalemmal receptors such as aminopeptidase N and dipeptidyl peptidase^11^.

The clinical presentation of COVID-19 is dominated by respiratory signs with less frequent occurrence of gastrointestinal symptoms. The virus invasion is not limited to these two organs, particularly considering that significant expression of ACE2 is detected in other tissues, including heart, kidney, endothelium and CNS^12^. Viral infection of the brain^13^ may have multiple neurological and psychiatric consequences, contributing to both the acute phase of disease and its potential sequelae Fig.^1^. The neurotropism has been well documented for several β-coronaviruses including SARS-CoV-1, MERS-CoV and the HEV 67 N virus of porcine hemagglutinating encephalomyelitis^14–19^. Arguably, the primary way for SARS-CoV-2 is associated with ACE2 expressed in neurones and in neuroglia^20–22^ The ACE2 expressing neural cells are found in the circumventricular organs, such as the subfornical organ, the paraventricular nucleus, the solitary tract and in the rostral ventrolateral medulla^21^. All these regions have little protection of the blood brain barrier (BBB) and all are involved in cardiovascular and respiratory regulation. The lack of BBB makes these CNS sites vulnerable in many pathologies, such as various types of systemic inflammation including sepsis-associated encephalopathy, neuroinfection with bacteria, viruses or parasites, stress and autoimmune encephalitis^23,24^. Microglial cells localised in CVO seems to be in a state of chronic activation in the attempt to limit the entry of circulating neurotoxic molecules or invasive agents into the parenchyma and to preserve cerebral homoeostasis^25^. The SARS-CoV-2, similarly to other respiratory viruses, could gain access to CNS through several routes, for example by migrating through axons of the olfactory nerve^26^. The intranasal infection of SARS-CoV-1 or MERS-CoV^27^ was shown to result in rapid spread of viral particles into the brain possibly through the olfactory bulb by a retrograde axonal transport; viruses replicating in the nasal cavity may utilise the direct link with the olfactory bulb to colonise the CNS. In this paradigm the virus is transported through the axons of olfactory bulb neurones with subsequent infection of the specific type of neuroglia the sustentacular cells of the olfactory bulb^28^. When the virus was administered intranasally in extremely low doses, only the CNS was disseminated^5^, strengthening the concept of an intrinsic neurotropism of coronaviruses. In rodents ablation of the olfactory bulb prevented viral spread following nasal infection^29^. Further support for the role of nasal-olfactory route comes from clinical observations according to which anosmia develops early in SARS-CoV-2 infected subjects^30^. The SARS-CoV-2 RNA was present for 20 or more days in oropharyngeal and nasopharyngeal secretion samples of 30% of COVID-19 survivors, suggesting that SARS-CoV-2 can linger for a long time at both upper and lower respiratory tract^31^.

**Fig. 1:**
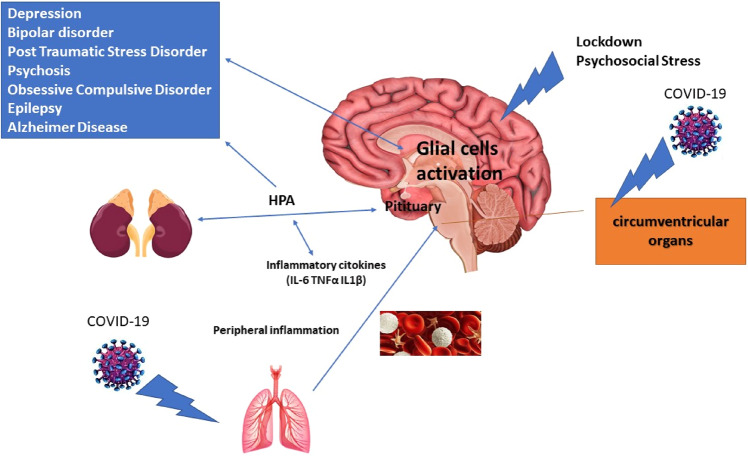
Neuropsychiatric sequelae of COVID-19. The SARS-COV-2 enters the body through various routes and causes systemic and tissue inflammation. Systemic inflammation compromises the blood-brain barrier (BBB) and floods the brain with pro-inflammatory factors. The virus may also cross the BBB at the level of the circumventricular organs or through retrograde axonal transport via olfactory bulb and infect the brain, thus instigating reactive gliosis, which leads to an increased production and secretion of cytokines and other pro-inflammatory factors. The combination of systemic inflammation, hypoxia resulting from respiratory failure and neuroinflammation may trigger or exacerbate psychiatric diseases.

The virus can also enter the brain through infecting endothelial cells lining brain vasculature; an electron-microscopic analysis of the frontal lobe identified SARS-COV-2 viral particles in the endothelium with some indications for virus transit to the neuropil^32^. The SARS-Cov-2 can enter the CNS using perivascular spaces of the glymphatic system^33^. Furthermore, viruses can invade the brain through other nerves, such as the trigeminal nerve, which projects nociceptive terminals to nasal cavities^34^. Similarly, sensory fibres of the vagus nerve, that innervate the respiratory tract, can present another invasion route^35^. Further evidence of the SARS-CoV-2 neuroinfection, oedema and neuronal degeneration were reported in post-mortem brain samples, while in a case of encephalitis genome sequencing confirmed viral presence in the cerebrospinal fluid^36^. Post-mortem analysis of nervous tissue from tissue of a 54 years-old man who died from severe respiratory failure associated with COVID-19 identified SARS-COV-2 viral particles in the olfactory nerve, in the gyrus rectus and in the brainstem with signs of profound damage to all elements of the tissue including glial cells, neurones, their axons and myelin^37^.

It seems therefore that SARS-CoV-2, similarly to SARS-CoV-1 and MERS-CoV infects the brainstem, in which the respiratory neuronal circuits are located, and, by analogy, a similar infection could occur and contribute to the respiratory failure, observed in SARS-CoV-2 pneumonia. Breathing depends on a central pattern generator located in the dorsolateral pons, in the nucleus of the solitary tract, and in ventrolateral medulla; this patter generator is responsible for respiratory rhythms and control of motor neurones innervating respiratory muscles^38,39^. In a sub-population of COVID-19 patients’ respiratory failure is manifested by a decreased breathing rate with hypoxia and hypercapnia. Many of these patients remain in a coma for days, despite the suspension of sedative treatment and the absence of apparent metabolic alteration, indicating viral encephalitis, which often is resolved without major sequelae^40^. This is not always the case, however, and when the extent of respiratory failure is overwhelming, patients die before the virus-induced brain damage can become evident^41^. Given the viral load in the brain stem, the subsequent reduction of ACE2 expression associated with the neuronal death, could lead to an alteration in baroreceptors function associated with an increase in the sympathetic tone and a severe, life threatening rise in blood pressure^42,43^. The encephalitis, reported as a complication of coronavirus infection, invariably affects not only the brain stem but also thalamus and white matter^26,44,45^. These aspects have to be taken in account by clinicians dealing with COVID-19 patients displaying severe cardiovascular and respiratory failure. Recognizing that respiratory symptoms may, at least in part, originate from the encephalitic damage to the brain stem, may help to design more effective treatments.

The damage to the brain stem as well as to other brain structures can also result from systemic inflammation, often referred to as systemic inflammatory response syndrome or “cytokine storm”^46,47^. At the same time the brain is a target for infectious toxic encephalopathy, associated with systemic toxaemia or hypoxia that accompany acute infectious diseases. Toxic encephalopathies have massive neurological and psychiatric presentations and even cerebral oedema, which however develops without accumulation of inflammatory markers in cerebrospinal fluid^48^. In addition, systemic infection and high levels of circulating cytokines often damage microcirculation, inducing oedema and thrombosis; the tromboembolia being reported in up to 30% of patients^49^. The cytokines, furthermore, activate autonomic nerves and hypothalamic-pituitary-adrenal axis which affect blood pressure. All these factors together stipulate ischaemic damage to the brain and are associated with occurrence of strokes which further increase mortality in COVID-19 patients^50^.

## Neuroinflammation in COVID-19

Despite the existence of BBB, the brain and the spinal cord communicate with the peripheral immune system, and hence every systemic inflammation affects the CNS^51^. In the context of COVID-19 the damage to BBB mediated by a massive increase in circulating pro-inflammatory factors is highly likely^52^. Compromised BBB allows an inflammatory storm to engulf CNS leading to functional damage. Once in the brain, peripheral inflammatory molecules as well as inflammatory cells instigate neuroinflammation thus perturbing homoeostasis, altering neural networks and inducing neuronal death^53,54^.

In the initial phases of systemic inflammation, antiviral immunity can effectively blunt viral dissemination, since reactivity of neuroglia and influx of surveying T cells can remove infectious elements, preventing spread without any further tissue damage^55^. In severe COVID-19, substantial release of chemokines and interleukins associated with systemic inflammation and the marked lymphopenia allow higher and a more prolonged persistence of a viral load; consequently deficient clearance of the virus together with the reactive gliosis can perpetuate neuroinflammation^56–58^. Even in mild cases, SARS-CoV-2 pneumonia causes hypoxia, which on its own can trigger or exacerbate inflammatory response of the CNS. Cerebral hypoxia activates key inflammatory transcription factors, including NF-κB and hypoxia inducible factor which stimulate overproduction of pro-inflammatory messengers^59^, trigger glial reactivity^60,61^, induce mitochondrial oxidative damage, and activate promoter region of numerous miRNAs, crucial for regulating gene expression during inflammation^62^. An excessive glial reactivity due to persistent exposure to pro-inflammatory cytokines also contributes to synapse loss and neuronal death^63,64^.

The impact of SARS-COV-2 infection on the brain is associated with excessive physical and psychological stress that stimulates the hypothalamic-pituitary-adrenal axis thus further exacerbating neuroinflammatory status^65^. The duration and frequency of exposure to stressors impacts neuroinflammation. In this sense while a response to short and moderate stressors could be beneficial, repeated or extended exposure to strong stressors exacerbates inflammation^66^. Exposure to long-lasting stress enhances inflammatory response through the release of several pro-inflammatory factors, which trigger down-stream signalling pathways, including NF-κB-dependent transcription. Contribution of glucocorticoids, associated with stress response, to sustaining and promoting neuroinflammation is complex, going beyond effects deriving from the activation of the signals downstream of their receptors^67^. Microarray experiments demonstrated that glucocorticoids drive expression of specific gene profiles, whereas concurrent co-activation of glucocorticoid receptors and NF-κB-dependent transcription induces a peculiar pattern of gene expression different from the one resulting from separate activation of each signalling pathway^68^. Neuroinflammation is a significant aetiological factor for a large number of neuropsychiatric and neuro-cognitive diseases, including neurodegenerative disorders^69–71^, depression^72^, psychosis^73^, autism^74^, drug abuse^75^, sleep disorders^76^ and epilepsy^77^. The neuropsychiatric burden of this pandemic is currently unknown, but is likely to be considerable. Based on the results from investigations of recent epidemics by corona respiratory viruses, SARS-COV-1 and MERS-COV, it is possible to assume that a significant percentage of subjects recovering from pneumonia do not fully regain their previous emotional state and cognitive abilities. Indeed, a study of neuropsychiatric consequences of SARS-COV-1 performed at 30–50 months after the infection demonstrated an occurrence of 40% of post-traumatic stress disorder (PTSD), 36.4% of depression, 15.6% of obsessive convulsive disorder, and an equal incidence for anxiety disorders^78^. Furthermore, a meta-analysis among SARS-COV-1 patients of mixed conditions showed neurocognitive deficits up to 18 months post-discharge^79^, including mild cognitive impairment^80^. Given this evidence the burden of long-term post-SARS-CoV-2 delirium and dementia may be notable, especially for elderly subjects who are more vulnerable to post-infectious neurocognitive sequelae. The average age of the subjects with severe COVID-19 is around 63 years, whereas patients under the age of fifty represent only 26% of all clinical cases. The ageing itself is the major risk factor for cognitive pathologies and neurodegeneration; severe systemic diseases as well as stress are known to provoke or accelerate cognitive decline in elderly. In the aged brain the neurogenesis is dwindling, synaptic plasticity deteriorates, metabolism is reduced and overall brain vulnerability to exogenous insults is increased. Ageing of the human brain is also associated with degeneration and atrophy of microglia and astrocytes which diminishes homoeostatic and neuroprotective support and again increases brain susceptibility to pathology^81–84^. Infection with SARS-CoV-2 (even in moderate clinical cases) thus promotes cognitive disorders with emergence of delirium, acute psychosis, exacerbation of mild cognitive impairment or with accelerating of dementia associated with various neurodegenerative conditions, including Alzheimer’s disease (AD)^85,86^. Conceptually, neuroinflammation contributes to the pathological development of neurodegeneration and often is considered as a common, even unifying feature of neurodegeneration^87^, while brain infection and ischaemic insults by themselves can trigger neurodegenerative process and instigate dementia^88^.

It is a truth universally acknowledged that systemic inflammatory challenge accelerates cognitive impairment, which implicates that the infection itself, as well as aberrations of the innate immune system, is responsible for the cognitive deficits^89^. Epidemiological observations as well as neuropathological analysis support the notion of a direct correlation between systemic infections, neuroinflammation and cognitive disorders, such as delirium and AD^90,91^. In this context, cohort studies identified pneumonia as the pre-eminent pathology responsible for hastening and boosting cognitive decline^92^. At the same time vaccinations against bacteria or viruses reduce the risk of the progressive evolution of dementia^93^. Close correlation between pneumonia and delirium in the elderly is a long-standing observation, and delirium, which represents the most common event of acute brain dysfunction, is a frequent complication of COV-19 clinical progression, perhaps due to the neurovirulence, severe peripheral inflammation, profound stress; even “social distancing” and loneliness which elderly experience during pandemic contribute to psychotic episodes^94^.

## COVID-19 and major depression

Systemic and tissue immune response contribute to the pathophysiology of numerous neuropsychiatric diseases through modifying neurochemical environment, synaptic transmission and plasticity, synthesis and secretion of neurotrophic factors, neurogenesis, and brain connectome. In this context, the major depression disorder (MDD) is one of the most frequent neuropsychiatric disorders linked to inflammatory injury to the brain. A large body of evidence has associated depression symptoms to pro-inflammatory factors^95^ and neuroglial failure^96^. This link specifically applies to subtypes of depression occurring in the elderly. Ageing substantially affects the levels and the activity of pro-inflammatory cytokines in the CNS. Systemic infection can itself trigger major depression in elderly patients, because of age-dependent decrease of immune homoeostasis^97^. In particular increased serum levels of interleukin-1β directly correlate with emergence of late life MDD^98^. Similarly, a correlation has been observed between inflammatory factors and some specific symptoms, for example, high levels of TNF-α and IL-2 associate with apathy and motor inhibition, whereas IL-6 associates with anhedonia and suicidality^99^. The levels of cytokines decrease when patients recover normal mood levels; conversely cytokines remain elevated in patients resistant to treatment^100,101^.

Severe cases of COVID-19 are almost invariably accompanied with excessive host immune response, mainly characterised by a massive increase in plasma levels of IL-6, which directly correlates with an unfavourable outcome of the disease^102^. At the same time abnormally high concentrations of IL-6 were detected in the cerebral spinal fluid of suicide attempters^103^, of subjects suffering from either depression or schizophrenia^104^, of old depressed patients^105,106^ and of mothers with post-partum depression^107^. A large body of evidence demonstrated that changes in IL-6 levels, both in plasma and in the brain, are implicated in the emergence of depression, although other factors, environmental or genetic in nature, provide an important contribution^108^. In the CNS IL-6 acts as a pro-inflammatory mediator, which promotes synthesis and secretion of additional inflammatory factors and acute phase proteins by astrocytes and microglia^109^. Thus IL-6, together with TNF-α and IL-1β, can be considered as one of the primary regulators of the immune response in the brain, while astrocytes and microglia are the major responders to IL-6, as well as prominent producers of IL-6 stimulated by damage and pathogen-associated molecular patterns (including viruses and their components), neurotransmitters and pro-inflammatory messengers^63,110–113^. Physiological plasma levels of IL-6 in adults range between 1–10 pg/ml whereas in a systemic inflammation is raises to several ng/ml;^114^ and even higher concentrations were reported for COVID 19^115^. Incidentally, high levels of IL-6 have been detected in the plasma, in the cerebrospinal fluid (CSF) and in the post-mortem prefrontal cortex of subjects with suicidal ideation, with non-fatal suicide attempts or committed suicides^116^. At the same time no direct correlation was found between plasma and CSF concentrations of IL-6 in subjects with suicide attempts, nor such correlation was detected for scores of depression severity^117^. The IL-6 levels in circulation also correlated with suicide endophenotypic behaviours, such as personality trait disorders, aggressivity and impulsivity^118^. This is in agreement with numerous findings proving the role of cytokines in regulating emotions and behaviours through interacting with specific brain areas and different neuronal pathways^119^. COVID-19 pandemic resulted in significant changes in lifestyle and interpersonal relationships condemning many to prolonged loneliness. These conditions of psychosocial stress can also have a detrimental effect on the most fragile subjects affecting their ability to modulate emotions^120^. Decreased control over impulsivity and feelings of fear in combination with inflammatory challenges to the brain might increase the risk of suicide.

## COVID-19 and bipolar disorder

Abnormal balance between the pro-inflammatory (IL-6 and TNF-α) and anti-inflammatory cytokines in the CNS and in the plasma have been repeatedly observed in patients with BD supporting the notion that neuroimmune response may be a prominent factor contributing to aetiopathogenesis of this illness^121,122^. In acute phases of BD either during manic or depressive episode, an activation of inflammatory cascades were reported, which was considered by many, but not by all, a characteristic feature of the acute illness, rather than a persistent trait of the disease^123^. Several cytokines, such as IL-1β, TNF-α, IL-6, interferon-γ were found to increase in circulation in acute phases of BD, with a parallel reduction in the anti-inflammatory factors IL-10 and transforming growth factor β-1, especially in the manic phase^123,124^.

Analysis of the presence of pro-inflammatory molecules in the CSF in BD patients revealed contradictory results. Somewhat high CSF levels of IL-8, monocyte chemoattractant protein 1(MCP-1 / CCL-2), and neurofilament light chain were detected in BD subjects, although these biomarkers did not correlate with the outcome of the disease^125^. A meta-analysis of CSF cytokines content in BD subjects revealed increased levels of IL-1β, the IL-8 showed statistically insignificant rise and no changes for IL-6 were detected^126^. The disparity between the levels of interleukins and chemokines in blood serum and in the CSF is another controversial issue. It is of course tempting to assume that this discrepancy is lost in COVID-19 since the overload of interleukins and chemokines, compromised BBB and activation of CNS resident and invading immune cells exacerbates the neuroinflammation and promotes a bidirectional flow of inflammatory messengers through a permeable barrier. Such a scenario, however, remains highly hypothetic and much more investigations and analysis are needed to reveal possible association of viral infection in general and COVID-19 in particular with BD.

## COVID-19 and reactive psychosis

A wide spectrum of immune system dysregulations as well as infections (together of course with genetic vulnerability, abnormalities in neurotransmission, stress and exposure to environmental factors such as childhood maltreatment) are recognised as potential pathogenetic factors of reactive psychosis^127^. Enhanced inflammation in psychosis has been confirmed by meta-analyses showing increased concentrations of cytokines and their receptors in chronic schizophrenia, as well as in drug-naïve patients in their first episode of psychosis (FEP)^128^. A recent study aimed at investigating pro-inflammatory cytokine profile in FEP patients showed an up-regulation of IL-6, TNF-α and IL-1β, which was not found in healthy siblings, suggesting familiar vulnerability is not involved in generating the inflammatory-related psychotic reactions^129^.

In an attempt to conceptualise the risk of the emergence of psychosis in subjects infected with SARS-COV-2, it should be emphasised that high levels of IL-6, correlate with reduced hippocampal size in schizophrenic subjects accounting, at least partially, for their cognitive deficits^130^. Moreover, elevated levels of IL-6 were detected in CSF of schizophrenic subjects^104^. Even more intriguing is the observation that high levels of IL-6 in adolescents correlate positively with the occurrence of psychosis later in life^131^.

## COVID-19 and obsessive-compulsive disorder

A growing body of literature reported the occurrence of obsessions and compulsions in patients who had recently recovered from viral encephalitis^132^. Already in 1930s more than a third of cases of obsessive and compulsive disorders (OCD) were recognised an organic in pathogenesis, and linked with Von Economo’s encephalitis^133^. Subsequently, neuropsychiatric literature was dotted with numerous case reports ranging from those of obsessive syndromes with post-encephalitic parkinsonism^134^, to those of post-encephalitic subjects in which diabetes insipidus coexisted with OCD^135^, to the six OCD patients with anamnesis of viral encephalitis^136^. Beside these early examples, more recently, high levels of Borna virus immune complexes and viral components (proteins, RNA) were detected in the blood and in peripheral mononuclear cells of OCD patients, reinforcing the notion of a significant link between viral infection and OCD in predisposed subjects^137^. In this scenario, since functional neuroimaging demonstrated OCD implies alterations in the striato-thalamo-cortical circuits, it was of interest that activity of these circuits may be affected in viral infection, possibly through interferences with glutamate transmission^138^.

Beyond any doubt immune dysfunction plays a causative role in childhood-onset OCD where the sudden onset of obsessive compulsive signs and tics occurs in the aftermath of a streptococcal infection, with subsequent production of auto-antibodies against neuronal antigens of the basal ganglia^139^, further supporting the notion that an alteration of the immune system may be implicated in the pathobiology of these disorders. Numerous investigations have shown a correlation between circulating pro-inflammatory cytokines levels and OCD^140,141^. Increase in blood concentrations of IL-1β, IL-6 and TNF-α in OCD patients was detected when compared to normal controls paired by gender, age, and educational level^142^. These findings are in agreement with results of studies investigating drug-naïve, comorbidity-free OCD subjects^143^. The observation that pro-inflammatory cytokines are increased in a study that eliminated any confounding factors, such as anxious or depressive comorbidity or the effects of psychotropic drugs, represents a more convincing support for the idea that immunological abnormalities contribute to the origin of OCD^141^. Systemic inflammation which is the prominent feature of COVID-19 may therefore trigger OCD in surviving subjects.

## COVID-19 and epilepsy

Extensive literature reports epilepsy and behavioural abnormalities as closely linked pathologies^144^. Indeed, psychiatric diseases are more frequent in epileptic subjects than in general population irrespective of the time of seizures onset, which could occur either before or after the appearance of psychiatric disorders, suggesting a mutual relationship and potentially shared aetiology^145^. This intriguing coexistence of psychiatric features in epileptic patients does not represent a coincidence or an ordinary comorbidity but more likely it reflects interconnected pathobiological processes^146^. Neuroinflammation may hint to the underlying mechanism shared by epilepsy and psychiatric disorders, albeit with distinct involvement of neuronal substrates^77^. This makes any rigid separation between epilepsy and some psychiatric disorders less stringent, and hence we included epilepsy in the discussion for the remarkable behavioural alterations and for the role of neuroinflammation in its pathogenesis.

The link between epilepsy and neuroinflammation is universally recognised^77^. Persistent neuroinflammatory cascade due to cytokine load and BBB damage is associated with glial reactivity, synaptic changes, and the generation of hyper-excitable networks with lower seizure threshold which all promote epileptic activity^147^. Epidemiological findings have indicated neuroinfection and systemic infections as one of major cause of acquired epilepsy^148,149^. Viral encephalitis, for example, increases the risk of subsequent seizures^149^. Increased concentrations of IL-1 were detected in plasma and CSF of different epileptic phenotypes, suggesting this cytokine seizure-inducing properties^150^. Changes in GABAergic-transmission and reduction of astrocytic glutamate uptake may account for IL-1 dependent increase in susceptibility to epilepsy^151,152^. Similarly, raised levels of IL-6 were reported in both plasma and CSF in patients suffering from a wide range of epileptic presentations, while this increased concentrations correlated with the severity of seizures^153^. Capability of IL-6 to promote epileptogenesis is further corroborated by the evidence that IL-6 overexpression induces abnormal ictogenesis in mice hippocampus^154^. Associations between epilepsy and COVID-19 have not yet been reported^155^, however, American Epilepsy Society already suggested that COVID-19 could increase the risk of sudden unexpected death in epilepsy (SUDEP). There are some reports which indicate that infections, bacterial or viral may increase the risk of SUDEP. At present, there are no data on the association between COVID −19 and SUDEP^156^.

## COVID-19 and post-traumatic stress disorder

Generally, albeit incorrectly, it is assumed that once the trauma is over and the subject is no longer under the pressure of stress, the path for steady recovery begins, since the time heals all wounds. Unfortunately, this is not always the case because in susceptible subjects the active stress instigates brain processes whereby traumatic memories suddenly re-emerge and disturb the mental health. The persistence of these conditions generates the PTSD^157^. The PTSD is no longer classified among anxiety disorders; it is considered a trauma or stress-related disorder^158^. The pathogenetic link between inflammation and PTSD is well documented^159^. Because of marked impact of stressors on the immune system, it is not surprising that PTSD is associated with the immune state^160^. Increased concentrations of pro-inflammatory factors were observed both within systemic circulation and in the brain in the context of PTSD^161^. Activation of neuroglia induced by heavy or persistent stressors can stimulate aberrant secretion of pro-inflammatory signals which could potentially facilitate the appearance of PTSD. Data from meta-analyses confirm a remarkable increase in pro-inflammatory molecules in subjects with PTSD, including IL-6, TNF-α, and IL-1β^162–164^. The levels of IL-10, an anti-inflammatory interleukin, have been also increased, probably in an attempt to offset the inflammatory processes triggered by stress, further highlighting a close link between inflammation, stress and PTSD^165^. The occurrence of PTSD was usually associated with occurrence of low-grade inflammation^166^. Beside changes in cytokines, PTSD is also connected with enhanced NF-κB expression, this transcription factor being implicated in the inflammation process and its elevated expression correlated directly with PTSD severity^167^. Moreover, PTDS is usually comorbid with depression as well as with anxiety, with drug addiction and with high frequency of suicide, since all these conditions share common inflammatory mechanisms into their pathogenetic processes^168^. However, it remains to be elucidated whether in all cases the relationship is mutual and what factors, along with the inflammatory ones, play a causative role in determining the comorbidities observed. The PTSD can be a likely outcome for COVID-19 sufferers. This stems not only from severity of systemic inflammation and viral invasion into the brain, but also from the gravity of stress caused by an unexpected pandemic which, for the high mortality, has a shocking value.

## Schizophrenia and viral infection

Significant number of psychotic episodes in the aftermath of Spanish flu pandemic has highlighted the possibility of increasing incidence of schizophrenic disorders in subjects infected with the SARS-COV-2^169^. High levels of coronavirus immunoreactivity in subjects with recent onset of psychotic episodes as well as the serious neuropsychiatric complications including auditory and visual hallucinations as well as severe delusions have been reported in COVID-19 patients^120,170,171^. Although neurodevelopmental origins of schizophrenia are generally accepted, other aetiological factors such as direct effect of a viral neuroinfection or indirect effect of immune aberrations occurring in adult subjects cannot be excluded^172^. Schizophrenia is also considered as a neurodegenerative illness in adulthood, with neuronal shrinkage and loss, oligodendrocyte damage, alterations in synaptic connectivity, all likely associated with cognitive impairments^162–173^. Although there are no evidence directly linking COVID-19 with the risk of schizophrenia, frequent occurrence of psychotic episodes highlights the need for further, more detailed investigations.

## Conclusions

The SARS-COV-2 pandemic poses a long-lasting challenge, which not only affects cardio-respiratory system but links systemic infection to neuropsychiatric diseases. Investigations of previous viral respiratory epidemics have demonstrated the onset of a wide range of psychiatric disorders over the course and in the aftermath of the infection. The pandemic of Spanish flu in 1918–1920 instigated speculation of the causative role of viral infection in the pathogenetic mechanism of behavioural disorders in bipolar and schizophrenic subjects. Karl Menninger was one of the first to declare that he was persuaded that dementia praecox (as schizophrenia was called in those days) is, in majority of instances, a somatopsychosis, “the psychic manifestations of an encephalitis”^174^. In the same period, Jacob Kasanin and J.W. Petersen suggested that “a thorough review of some of the early histories of atypical cases of schizophrenia or affective disorders may reveal a previous encephalitis”^175^.

At present, there are few preliminary studies considering neuropsychiatric complications of COVID-19, however, on the basis of the results of the previous epidemics of various respiratory viruses it is possible to assume an increased incidence of mental pathologies as an unwanted sequelae. Not only SARS-COV-2 can penetrate the brain and cause direct damage to neuronal networks, the experience of potentially lethal and untreatable COVID-19 is the cause of a severe distress, which may induce long term behavioural changes or aggravate a pre-existing mental illness. Here we outlined possible neuropsychiatric complications that could arise in subjects infected with SARS-COV-2. Patients with COVID-19 could present with a wide range of neuropsychiatric symptoms, which result from systemic inflammation, CNS effects of cytokines, infection of neural cells by SARS-COV-2, neuroinflammation, glial dysfunction or aberrant epigenetic modifications of stress-related genes. This review was intended to draw special attention to the psychiatric aspects of COVID-19, because minimizing their relevance by claiming that sometimes “an abnormal reaction to an abnormal situation is a normal behaviour”^176^ could be an unforgivable mistake.

## Footnotes

1 Holland, H., 1839, Medical Notes and Reflections, Haswell, Barrington, and Haswell, Philadelphia, p. 136.

2 Ravenholt, R. T., and Foege, W. H., 1982, 1918 influenza, encephalitis lethargica, parkinsonism, Lancet, v. 2, p. 863.
